# The in vitro antimicrobial activities of four endodontic sealers

**DOI:** 10.1186/s12903-019-0817-2

**Published:** 2019-06-18

**Authors:** Yuting Huang, Xiaoshuang Li, Preeti Mandal, Yan Wu, Lin Liu, Huihua Gui, Jiarong Liu

**Affiliations:** 1 Department of Stomatology, Union Hospital, Tongji Medical College, Huazhong University of Science and Technology, 1277# JieFang avenue, Wuhan, Hubei Province People’s Republic of China; 2 Department of Laboratory, Union Hospital, Tongji Medical College, Huazhong University of Science and Technology, Wuhan, People’s Republic of China; 3 Department of Cadre health care section, Union Hospital, Tongji Medical College, Huazhong University of Science and Technology, Wuhan, People’s Republic of China

**Keywords:** Antimicrobial activity, Root canal, Sealers

## Abstract

**Background:**

The purpose of this study was to investigate the antimicrobial activities of four endodontic sealers (GuttaFlow2, AH Plus, ProRoot MTA and RealSeal) against *E. feacalis*, *E.coli* and *C.albicans*.

**Methods:**

The antimicrobial activities of four endodontic sealers were assessed by both agar diffusion test (ADT) and direct contact test (DCT) in this study. In ADT, the results were reported as the diameter of the growth inhibition zone. Both fresh and 1-day-setting sealers were measured. In DCT, microorganisms in suspension were exposed to the sealers for 10, 30 and 60 min and the survival of microorganisms were determined after exposure at different time points(after mixing, 1 and 7 days). The number of colony-forming unit (CFU) was counted. The results were analyzed with ANOVA and Tukey tests.

**Results:**

Both ADT and DCT results showed that Guttaflow2 presented no effect against any tested microorganisms. In ADT, fresh RealSeal had the largest inhibition zone against all tested microbes, followed by AH Plus and ProRoot MTA. ProRoot MTA demonstrated inhibition zones against all the three test microbes after setting for 1 day, while the other three sealers showed no inhibition activity. In DCT, fresh AH Plus had the strongest antimicrobial effects against all the three tested microorganisms for every contact times, while its antimicrobial activity diminished significantly with time. Fresh RealSeal and ProRoot MTA also showed strong antimicrobial effect and still showed antimicrobial effect after 1-day-setting. The antibacterial effects of RealSeal against *E. faecalis* and antifungal effect of ProRoot MTA were observed after 7 days of setting.

**Conclusions:**

GuttaFlow2 had no antimicrobial activity. Freshly mixed RealSeal and AH Plus demonstrated strong antimicrobial effects. RealSeal showed antimicrobial effects after setting in DCT. ProRoot MTA showed high antimicrobial activity and exhibited anti-inflammation potentials after setting.

## Background

Microorganisms and their products are the main pathogenic factors in pulpal and periapical infection [1]. Therefore, to eliminate microbial agents from the infected root canal system is the chief aim of endodontic treatment [2, 3]. Instrumentation, irrigation and intra-canal medication in root canal treatment (RCT) process help to eliminate the infective substances [4, 5]. However, even after these procedures, some residual microbes still remain in the root canal system, which could be a potential source of inflammation [6]. Therefore the antimicrobial action of root canal sealers is also important in the successful outcome of RCT. Hence, it is valuable to investigate the antimicrobial activity of endodontic sealers.

Among several classes of endodontic sealers, antimicrobial activity of cold flowable filling system GuttaFlow2, epoxy resin-based AH Plus, calcium silicate–based MTA and multi-methacrylate resin-based RealSeal have been investigated in this study. GuttaFlow2, a silicone sealer, incorporates nanosilver as the antimicrobial component. Its higher biocompatibility has been elucidated in our former study [7]. Its antimicrobial effects on different microorganisms involved in root canal infections are yet to be elucidated.

AH Plus, an epoxy resin-based sealer with broad clinical applications, is accepted to be the golden standard against which all new sealers are compared with [8–10]. The antimicrobial activity of AH Plus has been widely investigated. Studies have showed that AH Plus inhibited the growth of both bacteria and fungi significantly [1, 11–13]. ProRoot MTA, a calcium silicate–based material with various clinical applications was chosen in the present study, as it is the bioceramic cement to which new root-end filling materials are being compared [14–16]. The antimicrobial effect of MTA has been reported but with controversial results [14–16]. Limited information was obtained regarding the antimicrobial activity of RealSeal sealer, a third generation of multi-methacrylate resin-based material, containing bioactive glass, calcium hydroxide, and radiopaque filler [17, 18].

There are various methodologies for evaluation of the antimicrobial activity of endodontic filling materials. In the present study, the antimicrobial activity of four endodontic sealers were evaluated by agar diffusion test (ADT) and direct contact test (DCT). ADT is one of the most commonly used techniques [11]. But the limitation of this method is its dependency on diffusion and physical properties of test materials. DCT evaluates the antimicrobial properties of the root canal sealers by counting the number of microbial colonies after plating on agar plates [9, 10, 14, 17]. Even insoluble materials can be tested with this quantitative assay. Therefore, the aim of the present study was to evaluate the antimicrobial activity of four different endodontic sealers including GuttaFlow2, AH Plus, ProRoot MTA and RealSeal against microorganisms commonly isolated from infected root canals (i.e., *E.faecalis*, *E.coli* and *C.albicans*) by both ADT and DCT.

## Methods

### Sealers

In this study, four endodontic sealers were tested: ROEKO GuttaFlow2 (Coltène Whaledent, GmBH+Co. KG, Langenau, Switzerland); AH Plus sealer (Dentsply DeTrey, Konstanz, Germany); gray ProRoot MTA sealer (Dentsply Maillefer, Ballaigues, Switzerland) and RealSeal sealer (SybronEndo, Orange, CA). The sealers were prepared in compliance with the manufacturer’s recommendations. For evaluating the antimicrobial activity of the sealers, ADT and modified DCT were carried out under strict aseptic precautions in superpurgative working table.

### Microorganisms

Antimicrobial activities of the sealers were evaluated against *E.coli* (AT-25922), *E.faecalis* and *C.albicans*. Specimens of *E.faecalis* and *C. albicans* used in this study were kindly granted from Clinical Laboratory, Union Hospital, Tongji Medical College, Huazhong University of Science and Technology. Tested microorganisms and culture medium are summarized in Table 1.

**Table 1:** Microorganisms and culture media used in this study

Microorganism	Culture medium	
Gram-positive cocci		
*Enterococcus faecalis*	LBb	LBa
Gram-negative rod		
*Escherichia coli*	LBb	LBa
Fungi		
*Candida albicans*	SDb	SDa

*LBb* Luria-Bertani broth, *LBa* Luria-Bertani agar, *SDb* Sabourauds broth, *SDa* Sabourauds agar

Tested microorganism was cultivated on blood agar at 37 °C for 48 h. In order to ensure purity, three to four colonies were picked up and resuspended in 5 mL broth. After cultured at 37 °C overnight, the inoculum was adjusted to match the turbidity equivalent to 0.5 McFarland Standard (approximately 1.5 × 10^8^ CFU/ml) for further investigation.

### Agar diffusion test

Agar diffusion test was conducted on double-layered plates. The base layer was made of 40 ml sterilized Luria-Bertani or Sabouraus agar. Four wells of 5 mm depth and 3 mm diameter were punched and the freshly mixed sealers were placed into the wells. Sealers that were placed and incubated at 37 °C for 24 h were considered as set samples. 0.5 ml McFarland scale of microbial suspensions was seeded into 15 ml of the Luria-Bertani or Sabouraus agar as the second layer. After incubating at 37 °C for 24 h, the diameter of the inhibition zones around each well were measured with a millimetre ruler with accuracy of 0.5 mm. The mean diameter of measured zone was analyzed statistically to assess antimicrobial activity of the tested sealers.

### Direct contact test

The modified DCT was performed to assess the antimicrobial properties of the endodontic sealers [19]. The endodontic sealers were applied in 96-well cell culture plates. The plate was held vertically and an equal amount (approximate 20 mg) of the test sealers were placed on the bottom of each well. The samples tested just after curing were designated as fresh group (group 1); The specimens that were allowed to set for 1 and 7 days in a humid atmosphere at 37 °C before testing were designated as 1-d and 7-d samples (groups 2 and 3). A 20 μL of microbial suspension (1.5 × 10^8^ CFU/mL) was carefully transferred to the surface of each sealer. The suspensions placed on the uncoated footwells were used as positive controls. Sealers incubated without microorganisms were used as negative controls. The plates were incubated at 37 °C for 10, 30, and 60 min and 180 μL of sterile saline was then added to each well. After being gently mixed with a pipette for 1 min, the microbial suspension from each well was transferred and serially diluted in sterile saline. To assess the survival of microbes, 20 μL aliquots were cultured onto LBa or SDa plates after 10-fold serial dilutions. The plates were then incubated for 48 h at 37 °C, colonies were counted, and the CFU/mL was calculated. The experiment was performed in duplicate.

### Controls for carryover effect

The carryover effect of tested sealers was done according to the methodology as described by Zhang et al. [10]. Equal amount of sealers as for DCT were placed on the footwells of 96-well plate. Twenty minutes after mixing, 20 μl of sterile saline solution was placed in direct contact with each specimen. The plates were incubated in 100% humidity at 37 °C for 1 h and 230 μl broth (LBb or SDb) was added to each well. The broth was gently mixed for 1 min and 20 μl of the mixed broth was transferred into 960 μl broth (LBb or SDb). A 20 μl of microbial suspension (1.5 × 10^8^ CFU/ml) was added into the first dilution tube at the same time. Sterile saline solution placed on the uncoated wells was used as positive control. Survival of microorganism was determined by using 10-fold serial dilution and inoculated onto agar plates. After incubation for 48 h at 37 °C, colonies on the plates were counted, and the CFU/mL was calculated. All experiments were performed at least twice. The presence of carryover effect was assessed by comparing the log^10^CFU/ml of each sealer with positive controls.

### Analysis

In ADT, the diameters of the inhibition zones were measured and recorded in each experimental group. The mean value and standard deviation (SD) of the diameters were analyzed by one-way ANOVA in SPSS, version 20. In DCT, the CFU counts were transformed to their log^10^ values. Data were confirmed to be normally distributed by using Kolmogorov-Smirnov normality tests. One-way variance analysis and tukey multiple comparisons were done to reveal the statistical significance in different group. Graph Pad Prism 5 was used to present the data in bar diagram form. *P*-values less than 0.05 were considered statistically significant.

## Results

### Agar diffusion test

**Fig. 1:**
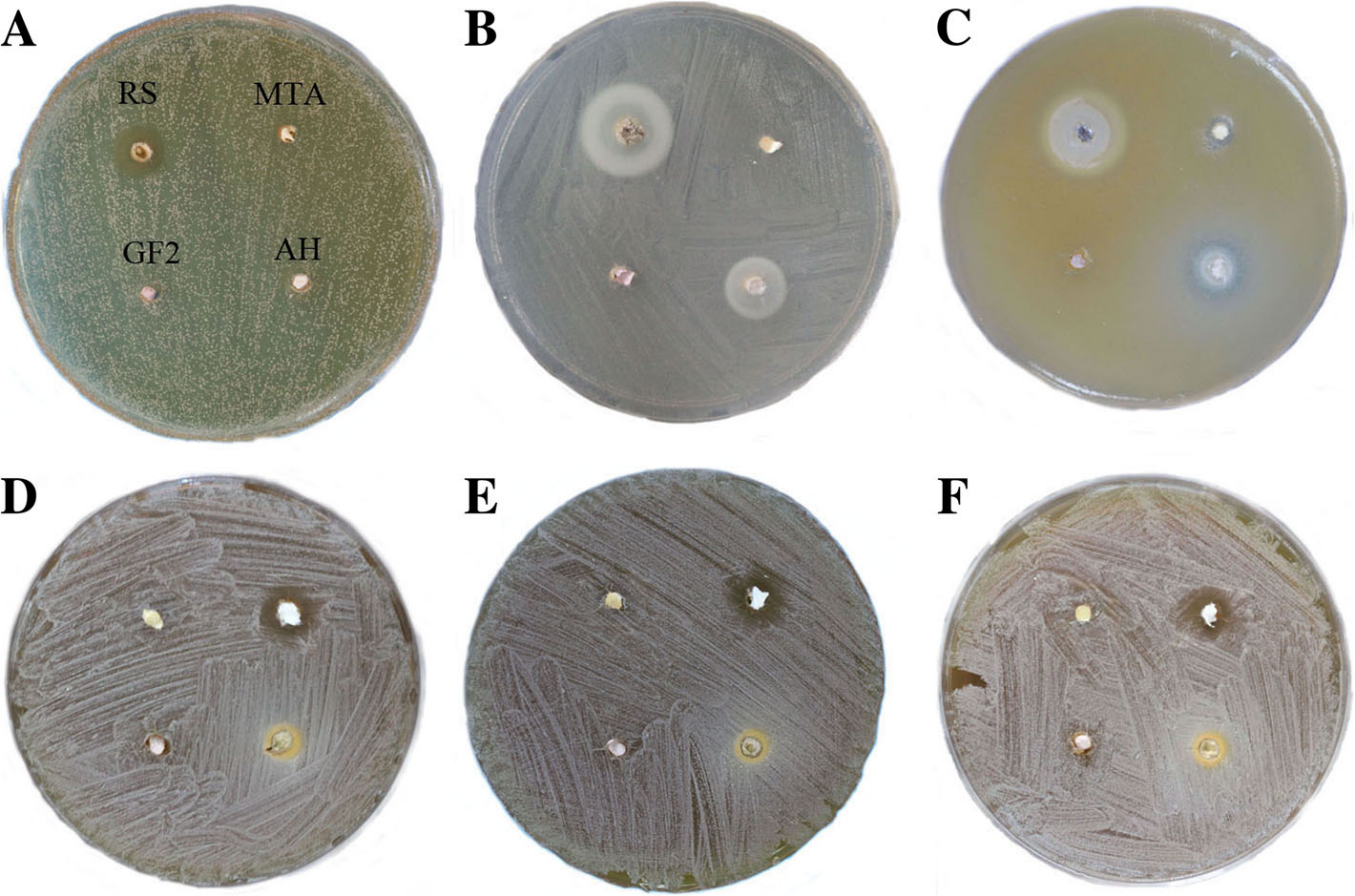
Zone of inhibition for four endodontic sealers. **a** Fresh sealers against *E.faecalis*; **b** Fresh sealers against *E.coli*; **c** Fresh sealers against *C.albicans*; **d** 1-day-setting sealers against *E.faecalis*; **e** 1-day-setting sealers against *E.coli*; **f** 1-day-setting sealers against *C.albicans*. GF2, GuttaFlow2; AH, AH Plus; RS, RealSeal; MTA, grey Pro Root MTA

The zones of microbial growth inhibition from ADT are presented in Fig. 1 and Table 2. GuttaFlow2 didn’t demonstrate any inhibition zones in either fresh or set samples. Fresh RealSeal had the largest inhibition zone against all the three test microbes, which is significantly larger than the other sealers (*p* < 0.01). Fresh AH Plus showed larger antimicrobial activity against *E.coli* and *C.albicans*, but no inhibition activity against *E.feacalis*. Fresh ProRoot MTA showed a slight inhibition against *C.albicans*. After incubation for 24 h, ProRoot MTA demonstrated inhibition zones against all the three test microbes, while the other three sealers showed no inhibition activity.

**Table 2:** Means and standard deviation of zones of inhibition for ADT (mm)

Sealers Group	Microorganisms		
	*E.faecalis*	*E.coli*	*C.albicans*
Fresh-GuttaFlow 2	0	0	0
Fresh-AH Plus	0	3.17 ± 0.29	3 ± 0
Fresh-RealSeal	4.83 ± 0.29	15.17 ± 0.76	8 ± 1
Fresh-MTA	0	0	3.5 ± 0.5
1 day-GuttaFlow 2	0	0	0
1 day-AH Plus	0	0	0
1 day-RealSal	0	0	0
1 day-MTA	8 ± 0	9.3 ± 0.29	7.3 ± 0.29

### Direct contact test

**Fig. 2:**
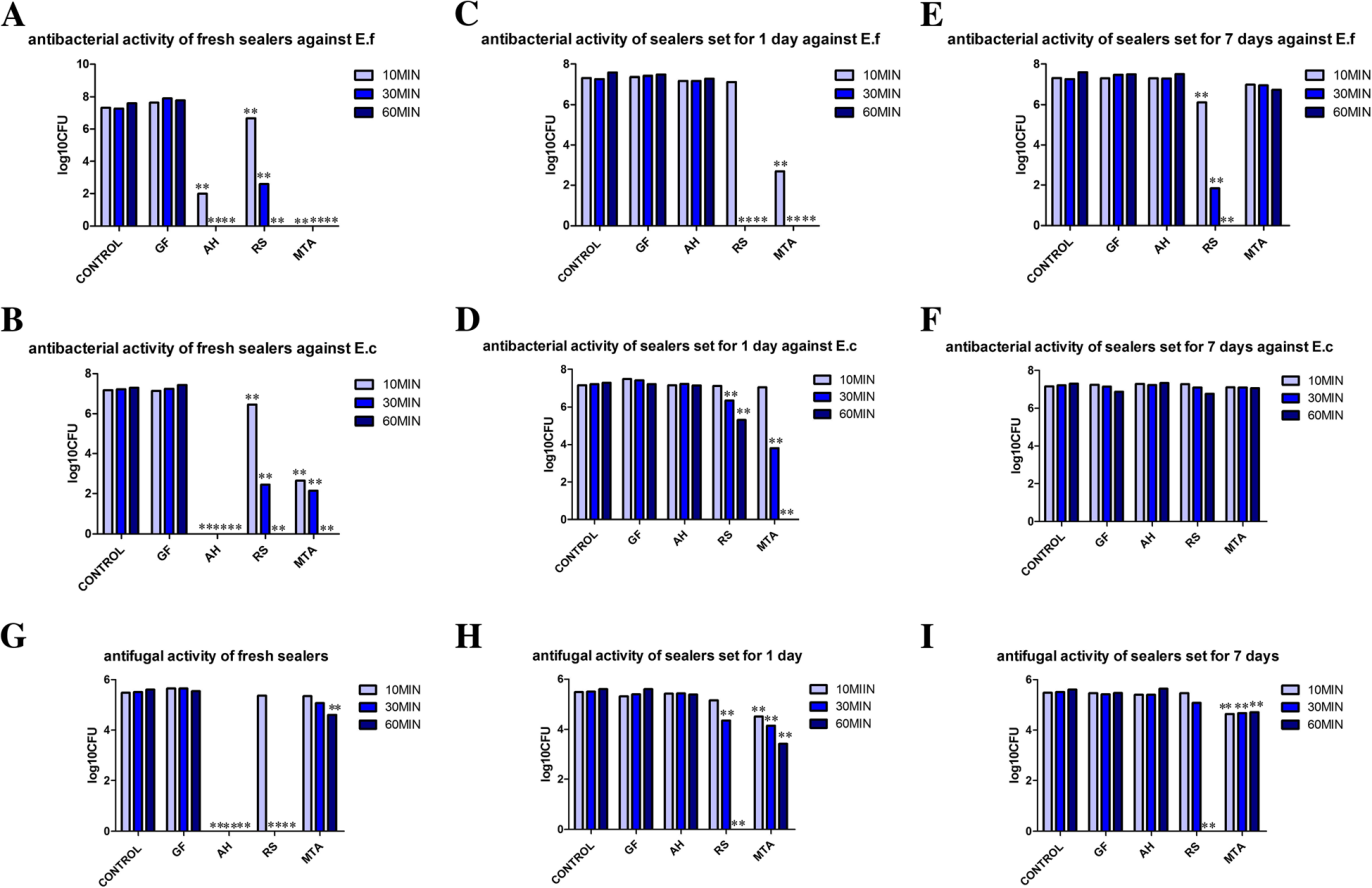
Survival of microbes after direct contact test for 10, 30 and 60 min with different sealers. Growth of *E. faecalis* after being in contact with fresh (**a**), one-day-set (**c**) or 7-day-set (**e**) sealers. Survival of *E.coli* after being in contact with fresh (**b**), one-day-set (**d**) or 7-day-set (**f**) sealers. Survival of *C.albicans* after being in contact with fresh (**g**), one-day-set (**h**) or 7-day-set (**i**) sealers. Bacterial suspension placed on uncoated wells was used as the control. The survival of bacteria was assessed by culturing aliquots of 20 μL into LBa or SDa plates after 10-fold serial dilutions. Colonies on the plates were counted after 48 h incubation and CFU/mL was calculated. All experiments were performed in duplicate. GF2, GuttaFlow2; AH, AH Plus; RS, RealSeal; MTA, grey Pro Root MTA; E.f, *Enterococcus faecalis*; E.c, *Escherichia coli*

The results of antimicrobial activity of tested endodontic sealers from modified DCT are presented in Fig. 2. No significant difference was found between GuttaFlow2 and positive control against the tested microbes at all tested time points (*p* > 0.05). As for fresh sealers, AH Plus had the strongest antimicrobial effects. It demonstrated significant inhibition against *E.faecalis*, *E.coli* and *C.albicans* for every contact times considered. Freshly mixed RealSeal and ProRoot MTA also showed strong antimicrobial effect against the test microbes. The antimicrobial effect of AH Plus diminished significantly over time. It didn’t show any antimicrobial effect after setting for 1 and 7 days. ProRoot MTA showed antimicrobial effect against the microbes after 1-day-setting at all tested time points and RealSeal demonstrated antimicrobial effect at most time points. After 7 days of setting, the antibacterial effects of RealSeal against *E. faecalis* strains for every contact times were significantly greater than other sealers. Antifungal effects were observed in ProRoot MTA and RealSeal with samples set for 7 days.

### Carryover effect control

Carryover effect was detected in the test of fresh ProRoot MTA against *E.coli* and *E.feacalis*, with significant differences compared to the positive control (*P* < 0.05).

## Discussion

The main purpose of endodontic sealers in RCT is to fill the gap between the core material and root canal wall, and help minimize leakage to reduce the possibility of infection by residual microbes. Good antimicrobial property is considered to be highly desirable for an ideal root canal sealer. The present study reported the antimicrobial activities of four endodontic sealers: GuttaFlow2, AH Plus, RealSeal and ProRoot MTA.

The result demonstrated that GuttaFlow2 did not show any antimicrobial activity in both ADT and DCT. GuttaFlow incorporates silver nanomaterials as its antimicrobial material and it is expected to exhibit broad-spectrum biocidal activity toward bacteria, fungi and viruses [18]. Several factors, such as particle size, shape, stability, and water chemistry could influence the antibacterial effects of silver nanomaterials [20]. GuttaFlow2 incorporates micro-silver as the antimicrobial component instead of nano-silver in GuttaFlow. Micro-silver has larger size and smaller specific surface area compared to nano-sliver, which leave a lower number of atoms exposed to the surface available for biochemical reaction with microbes and results in lower antimicrobial activity. Similarly, previous studies have revealed a minimal antibacterial effect of GuttaFlow, the foremost formulation of GuttaFlow2 [1, 8, 20, 21]. Wainstein et al.evaluated the antibacterial activity of GuttaFlow2, epoxy resin-based (AH Plus) and zinc oxide and eugenol-based (Endofill) sealers against *E.faecalis* [22]. The result showed that the modifications in silver particle size of GuttaFlow2 did not result in antibacterial effect.

AH Plus is a polymeric material. In the present study, fresh AH Plus demonstrated high antimicrobial activity. Studies have shown that epoxy-resin sealers demonstrated similar antimicrobial properties [9, 23, 24]. Spangberg et al. firstly reported that all these sealers released formaldehyde in the polymerization process [25]. Other study stated that the antimicrobial effect of resin-based sealers might also be associated with bisphenol A diglycidyl ether [26]. The unpolymerized components (ie, epoxide and amine) may be released into the surrounding milieu during the setting process, which might also explain the initial strong antimicrobial activity [24]. The antimicrobial effect of AH Plus diminished significantly with time. After 1 and 7 days setting, the sealer cured completely and the decreased antibacterial effect of the set specimen may be caused by the reduction in these antimicrobial substances.

Similarly, other previous studies [8–10, 24] had also supported the antimicrobial behavior of AH Plus. Kangarlou et al demonstrated that freshly mixed AH Plus had strong antimicrobial activity against *E.faecalis*, while no antimicrobial activity was found after 1, 3 and 7 days setting [27]. Zhang et al also reported that AH Plus sealer lost most of their antimicrobial activities after 24 h [10]. The result of this study is consistent with that of the former studies.

MTA was firstly introduced as root end filling material in 1993 [28]. Because of its bioactivity and biocompatibility, MTA is widely used for various endodontic purposes. Several previous studies have proved the good antibacterial activity of MTA [9, 29]. In this study, ProRoot MTA demonstrated inhibition zones against all the three test microbes after 24 h in ADT. In DCT, freshly mixed and 1-day-setting ProRoot MTA showed strong antimicrobial effect against all test microbes. After setting for 7 days, only antifungal effect was observed in MTA. Calcium hydroxide is the main chemical component released from MTA during the polymerization reaction and this resulted in increasing pH throughout a period of time [30–33]. The increased pH after setting could explain the antimicrobial behavior of MTA in this study.

A study by Koruyucu et al showed that MTA had significantly higher antibacterial activity than control in freshly mixed and 1-week samples [34], which is consistent with the current study. A previous study demonstrated that MTA Fillapex had antibacterial effect on *E. faecalis* when freshly mixed, while it lost this property after setting [15]. The findings of the present study did not coincide with the study above. The antimicrobial properties of MTA depends on multiple factors such as bacterial types, study duration, use of a fresh or cured material, the use of direct contact or extract [35]. Different survey methods and polymerization situation may be the main reasons for the incongruence between our findings and those of other studies.

RealSeal sealer, is a new generation of Resilon/Epiphany system, which consists of a self-adhesive resin-based sealer and Resilon. In the present study, freshly mixed RealSeal showed significant antimicrobial effect against three tested microorganisms in both ADT and DCT. In ADT, set RealSeal samples did not inhibit the growth of tested microbes. In DCT, the antibacterial activity of set samples against *E.coli* diminished with time significantly. While its antimicrobial effect against *E.feacalis* and *C.albicans* remained after setting for 1 and 7 days and demonstrated anti-inflammation potentials after setting. The antimicrobial activity was influenced by several factors in the setting process of RealSeal sealer. Self-etching primer in RealSeal may decrease the environment pH in the process of setting [36]. The acidic pH levels may affect the antimicrobial effect and influence the diffusion rate of its components [36], this may lead to the rapid reduction of antimicrobial activity in RealSeal system. The uncured monomer leaching from the resin in RealSeal may also play an important role in the antimicrobial activity.

Available information about the antimicrobial activity of RealSeal was limited. Mohammadi et al. reported that RealSeal had significantly greater effect against *Streptococcus mutans* than GuttaFlow [18], while Faria-junior et al.found that sealers set for 2 and 7 days had no antibiofilm activity [37]. The conflicting findings of antimicrobial activity of RealSeal may be influenced by the tested microbes, different experimental methods and polymerization situation.

Carry-over effect of the sealers can cause the growth inhibition of tested micro-organisms in DCT and lead to a false negative result [15]. In the present study, Carryover effect was detected in the test of fresh mixed ProRoot MTA against *E.coli* and *E.feacalis*, with significant differences compared to the positive control. The result is different with former findings [15].

## Conclusion

Freshly mixed AH Plus and RealSeal demonstrated strong antimicrobial effects. Antimicrobial activity of RealSeal was conflicting in different tested methods. ProRoot MTA exhibited antimicrobial potentials after setting. GuttaFlow2 had no antimicrobial activity. Therefore, further modifications are suggested be made to improve the antimicrobial activity of GuttaFlow2.

## Data Availability

The data sets used and/or analyzed during the current study available from the corresponding author on reasonable request.

## Abbreviations

ADT: Agar diffusion test
*C.albicans*: *Candidia albicans*
DCT: Direct contact test
*E.coli*: *Escherichia coli*
*E.faecalis*: *Enterococcus faecalis*
MTA: Mineral trioxie aggregate
RCT: Root canal treatment
